# Insulin-Containing Wound Dressing Promotes Diabetic Wound Healing Through Stabilizing HIF-1α

**DOI:** 10.3389/fbioe.2020.592833

**Published:** 2020-12-18

**Authors:** Peilang Yang, Di Wang, Yan Shi, Mingzhong Li, Min Gao, Tianyi Yu, Dan Liu, Jie Zhang, Jizhuang Wang, Xiong Zhang, Yan Liu

**Affiliations:** ^1^Department of Burn and Plastic Surgery Ruijin Hospital Affiliated to Shanghai Jiao Tong University School of Medicine, Shanghai, China; ^2^Department of Anesthesiology, Shanghai Jiao Tong University Affiliated Sixth People’s Hospital, Shanghai, China; ^3^National Engineering Laboratory for Modern Silk, College of Textile and Clothing Engineering, Soochow University, Suzhou, China

**Keywords:** diabetes, wound healing, insulin, HIF-1α, angiogenesis

## Abstract

HIF-1α is seen as a major regulator during wound healing and controls many wound healing processes, such as angiogenesis, extracellular deposition, and reepithelialization. A diabetic state plays a vicious effect on wound healing, and the destabilization of HIF-1α is a non-negligible factor. Insulin-loaded silk fibroin microparticles were prepared to release insulin by covering the wounds, and this material was proven to promote wound healing in both *in vitro* and *in vivo* studies. In this work, we found that this insulin-containing wound dressing could accelerate diabetic wound healing by promoting reepithelialization, angiogenesis, and extracellular matrix, especially collagen deposition. Meanwhile, HIF-1α was stable and accumulated in insulin-containing dressing to group wound cells, which was significantly unstable in the control group. In further studies, we showed that methylglyoxal (MGO), the main form of advanced glycation end products (AGEs), accumulated significantly and caused the destabilization of HIF-1α in the diabetic state. Insulin could alleviate the MGO-induced HIF-1α unstable state and promote HIF-1α target gene expression and its downstream biological effect such as angiogenesis and wound extracellular matrix deposition.

## Introduction

Diabetic non-healing wound is one of the most common late complication of diabetic patients. The morbidity rate of diabetic non-healing wound ranges from 5 to 20% among all diabetic patients. Diabetic wound puts a huge pressure on the social health economic system for its retarded healing characteristic (Falanga, 2005; Davis et al., 2018). Histologically, diabetic wound shows the characteristics of impaired angiogenesis, reepithelialization, and extracellular matrix deposition (Demidova-Rice et al., 2012). All these characteristics are related to an unstable HIF-1α in the diabetic environment. HIF-1α is considered as the central regulator of wound healing, and it dominates wound healing processes, such as inflammation reaction, angiogenesis, reepithelialization, and extracellular matrix deposition (Hong et al., 2014).

In a diabetic wound environment, the stabilization of HIF-1α would be severely impaired in the hypoxia state, and the instability of HIF-1α is regarded as the major factor of diabetic non-healing wound. In the diabetic state, the instability of HIF-1α is often caused by the accumulation of advanced glycation end products (AGEs). Recently, methylglyoxal (MGO), the major form of AGEs, has been reported to induce HIF-1α instability in many ways (Ramasamy et al., 2006; Catrina, 2014). Therefore, many researches aim at stabilizing HIF-1α to improve wound healing (Mace et al., 2007; Botusan et al., 2008). Our previous research results show that insulin can act as a growth-like factor to promote wound healing by promoting angiogenesis, reepithelialization, and extracellular matrix deposition (Liu et al., 2009a, b). Based on these results, in this work, we develop a wound dressing by loading insulin into silk fibroin (SF) microparticles (Li et al., 2017). As many functions of insulin on wound healing overlapped with the functions of HIF-1α, we in this research intend to check the effects of insulin-containing dressing material on diabetic wound healing and whether insulin takes effect through regulating HIF-1α stabilization.

## Materials and Methods

### Reagents

Streptozocin (STZ), insulin for cell culture, and MGO were from Sigma-Aldrich (St. Louis, MO, United States). ProLong gold antifade mounting reagent with DAPI was purchased from Thermo Fisher Scientific (Carlsbad, CA, United States). RPMI 1640, DMEM, and phosphate buffer solution (PBS) were obtained from GIBCO (Carlsbad, CA, United States). Chemiluminescence reagent was from Millipore (Billerica, MA, United States). Insulin was from Roche chemical (Houston, TX, United States). OxiSelect MGO Competitive ELISA Kit was purchased from Cell Biolabs (San Diego, CA, United States). Matrigel was from BD Biosciences (Waltham, MA, United States).

The following antibodies were obtained from various suppliers: PECAM-1, collagen I, and collagen III were from Abcam (Cambridge Science Park, Cambridge); CBP, GLUT-1, VEGF-A, HIF-1α, and HRP-labeled secondary antibody were from Cell Signaling Technology (Danvers, MA, United States); and donkey anti-rabbit Alexa Fluor 488 was purchased from Thermo Fisher Scientific (Carlsbad, CA, United States).

### Animals

Forty male Sprague-Dawley rats, 6 weeks old and weighing 120–140 g, were purchased from Shanghai Laboratory Animal Center in the Chinese Academy of Sciences and housed at the Animal Science Center of Shanghai Jiao Tong University, School of Medicine (SJTUSM). The animals were maintained under a 12-h light/dark cycle at 22°C. Thirty rats were used to develop diabetes by a reported method. In brief, the rats were fasted for 16 h followed by a single dose of STZ (60 mg/kg BW dissolved in 0.1 mM citrate buffer) intraperitoneally (Junod et al., 1969). Rats were left to develop diabetes for 8 weeks. Random plasma glucose was measured, and a glucose level >300 mg/L was considered diabetic. The rest of the rats were set as normal control (Norm). The animal procedures were performed in accordance with the rules of the Animal Care Committee of SJTUSM, and all experiment protocols were approved by the SJTUSM Institutional Animal Care and Use Committee.

### Preparation of SF Microparticles

The preparation of SF microparticles with or without insulin was described in a previous study as follows (Li et al., 2017).

(1) Preparation of SF solution: Briefly, *Bombyx mori* silk fibers (Huzhou, China) were degummed three times in 0.05% Na_2_CO_3_ solution at 98–100°C for 30 min and dried at 60°C after thorough rinsing. The fibroin extract was dissolved in a ternary solvent of CaCl_2_:CH_3_CH_2_OH:H_2_O (1:2:8 molar ratio) at 72 ± 2°C for 1 h. The solution was then dialyzed in cellulose tubes (MWCO 9–14 kDa) in deionized water for 4 days.

(2) Preparation of SF microparticles: 100 mg insulin (27.5 IU/mg; from porcine, Wanbang Biochemical Pharmaceutical Co., Ltd., Xuzhou, China) was dissolved in 10 ml of 0.01 N HCl; then the pH of the solution was adjusted to 7.0 ± 0.1 using 0.1 N NaOH. The SF solution was diluted to 2.0 wt.% and mixed with glycerol at 30 wt.% of SF weight. The resulting insulin solution and SF solution were used as core and shell for coaxial electrostatic differentiation, respectively.

(3) Preparation of microparticle-loaded SF sponge dressing: The SF solution was diluted to 2.0 wt.%, and then 2-morpholinoethanesulfonic acid/*N*-hydroxysuccinimide/1-ethyl-3-(3-dimethylaminopropyl)carbodiimide hydrochloride (MES/NHS/EDC) was added to the SF solution at 20/10/20 wt.% of SF weight, respectively. Nine hundred microliters of the above mixed solution was added into a round aluminum box (*D* = 40 mm, *H* = 15 mm, *V* = 10 ml) and frozen at −80°C for 30 min. Then 10-mg SF microparticles were evenly laid on a frozen SF solution layer and frozen at −80°C for 15 min. On top of it, 700 μl of the above mixed solution was added to cover the microparticles and frozen at −80°C for 15 min, followed by 10-mg SF microparticles and 900 μl SF solution at a similar process. Then the SF mixture composite with two layers of microparticles was frozen at −80°C for 4 h and further freeze-dried to obtain microparticle-loaded SF sponge dressing. The dressings were sterilized with γ-ray irradiation and stored at 4°C.

### Scanning Electron Microscopy

The morphology of samples was observed by a scanning electron microscope (SEM, S-4800, Hitachi, Japan) described as before (Li et al., 2017). The size of microparticles was analyzed on the basis of SEM images with the Nano Measurer analysis software (Department of Chemistry, Fudan University, China. 2008 Jie Xu).

### *In vitro* Insulin Release

The *in vitro* insulin release was determined as described before (Zhao et al., 2012; Li et al., 2017). Insulin was labeled with fluorescein isothiocyanate (FITC) (Sigma-Aldrich). Three hundred microliters of FITC solution (10 mg/ml in dimethyl sulfoxide) was added to 10 ml of insulin solution (15 mg/ml in bicarbonate buffer, pH = 8.5, 0.1 M) and stirred at room temperature for 60 min. Next, 200 μl of 1 M hydroxylammonium chloride solution was added and stirred for 10 min at room temperature. The insulin was then purified using a 10 mm × 300 mm column with Sephadex G-50 equilibrated in 0.1 M sodium bicarbonate buffer (pH = 8.5) to remove any unreacted FITC. The fluorescent images were captured using an inverted fluorescence microscope (Olympus IX71, Japan). Physically absorbed microparticles were used as a control group. Ten milligrams of pure SF microparticles was directly immersed into 2 ml of FITC–insulin solution to adsorb insulin by permeation and physical absorption, which were named insulin-adsorbed microparticles. The remaining amount of insulin in solution was quantified to calculate the adsorption amount using a fluorescence spectrophotometer (FM4P TCSPC, Horiba Jobin Yvon), and the loading ratio in the insulin-adsorbed microparticles was about 5.5%. The insulin release profiles were determined by immersing 10 mg of microparticles and a sponge dressing containing 10 mg of microparticles in 10 ml of PBS (10 mM, pH 7.4). The samples were incubated in a water bath at 37°C with a 100-rpm shaking. One milliliter of medium was collected and replaced with an equal volume of fresh PBS after centrifugation at predetermined time points. The amount of released insulin was quantified using a fluorescence spectrophotometer.

### Wounding Procedure

One day before wounding, rats were anesthetized with a single intraperitoneal injection of thiopental sodium (40 mg/kg BW); the hairs on the back were shaved and thoroughly removed using the Nair hair remover lotion. On the day of wounding, the rats were anesthetized with thiopental sodium and a dose of tramadol. The animals’ backs were incised to produce a 1.5 cm × 1.5 cm wound.

In the first part, the rats were separated into two groups: the normal non-diabetic group (Norm) and the diabetic group (DM). In the second part, the rats were also separated into two groups: the group in which the diabetic wound was dressed with SF microparticles without insulin (Ctrl) and the group in which the diabetic wound was dressed with SF microparticles containing insulin (INS).

### Measurement of Wound Closure

Rats were anesthetized with thiopental sodium at the 5th and 11th days after wounding. The wounds were photographed and drawn on transparent tracing papers, and the papers were scanned. The wound sizes were analyzed using ImageJ software. The unhealed rate was calculated by comparing the unhealed wound area to the original wound area.

### Histological Observation

The wounds, including 5 mm of adjacent normal skin, along with the subcutaneous fat tissue were harvested at the 5th and 11th days after wounding. The tissue was fixed in 4% paraformaldehyde and embedded in paraffin. Sections with 6–7 μm thickness were stained with hematoxylin and eosin (H&E) for histological and morphometric observation and evaluation. Masson trichrome staining was used for collagen deposition evaluation.

### Immunohistochemistry

The wounds were harvested and fixed as mentioned above, and sections were then deparaffinized, rehydrated, and washed in distilled water. The sections were placed in 95∼98°C antigen retrieval citrate buffer in a container for 10–15 min. Endogenous peroxidase activity was blocked by placing the sections in 3% hydrogen peroxide in methanol for 10 min. Non-specific staining was blocked with normal goat serum, and the sections were incubated with anti-rat PECAM-1, VEGF-A, or HIF-1α overnight at 4°C. After washing, HRP-labeled secondary antibody was applied for 1 h at room temperature and then with diaminobenzidine and counterstained with hematoxylin.

### Immunofluorescence

Cells were washed with PBS three times and fixed with 4% paraformaldehyde in PBS at a pH of 7.4 for 5 min. The cells were incubated for 10 min with PBS containing 0.25% Triton X-100. Non-specific staining was blocked with normal goat serum, and the sections were incubated with anti-human HIF-1α overnight at 4°C. After washing, Alexa Fluor 488-labeled secondary antibody was applied for 1 h at room temperature. Coverslips were mounted with a drop of ProLong gold antifade mounting reagent with DAPI and sealed with nail polish to prevent drying and movement under the microscope.

### Western Blot

The tissues were homogenized by pulverization in liquid nitrogen and transferred to tissue lysis buffer with a protease inhibitor cocktail following centrifugation at 12,000 rpm for 15 min. The supernatants were removed and stored at −80°C. An equal amount of protein per lane (50 μg) was separated by 5–12% SDS-PAGE and transferred to a polyvinylidene difluoride membrane. The membranes were blocked by 5% non-fat powdered milk in Tris–buffered saline with Tween-20 (TBST) and then incubated with anti-PECAM-1, collagen I, collagen III, VEGF-A, CBP, GLUT-1, and HIF-1α primary antibody in 5% non-fat milk in TBST overnight at 4°C. The membrane was then washed extensively with TBST and then incubated with the secondary antibody for 1 h at room temperature. Bands were visualized with enhanced chemiluminescence. Relative quantities of protein were determined using a densitometer and presented in comparison with β-actin expression.

### Cell Culture

HUVECs and fibroblasts were purchased from ATCC and cultured as recommended.

### HUVEC Tube Formation Assay

The tube formation assays were operated according to the manufacturer’s instruction. Briefly, the Matrigel was thawed overnight on ice at 4°C, and the vial was swirled to ensure that the material is evenly dispersed when the Matrigel thawed. Chilled Matrigel (10 mg/ml) was added at 0.3 ml per well with pre-cooled tips, and the entire growth surface of 24-well culture plates were covered completely and evenly. Plates were incubated at 37°C for 45 min. The remaining liquid was carefully removed from the culture well without disturbing the layer of Matrigel just before use. Passage 4 HUVECs were detached, and the cells were resuspended in culture medium with 5 serum at 1 × 10^6^ cells/ml with or without 800 μM MGO or with 800 μM MGO and 10^–6^ M insulin. Into each well was added 300 μl of the cell suspension, and the angiogenesis assay plate was incubated for 12 h at 37°C, at 5% CO_2_ atmosphere. Cells were monitored and photographed using an inverted microscope.

### Statistical Analysis

Data analysis was performed using GraphPad Prism software (GraphPad Software Inc). *T*-tests were used to determine the significance of pairwise differences between means, unpaired *T*-tests was used for comparison between two groups, and one-way ANOVA (Dunnett’s *post hoc* test) was used to determine significance between means of several groups. Data satisfying the assumptions of ANOVA were verified before performing the tests. A *p*-value less than 0.05 was considered significant statistically, and a *p*-value less than 0.01 was considered statistically highly significant. Data are shown as mean ± standard deviation (SD).

## Results

### Diabetic Wounds Showed Significantly Retarded Healing Characteristics

**FIGURE 1:**
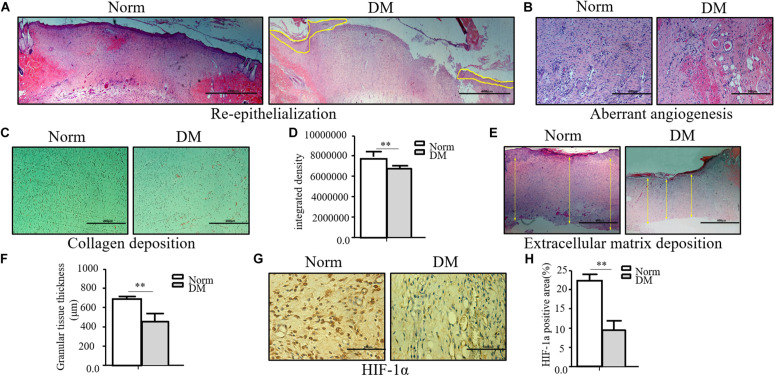
The wound healing process was significantly impaired in the diabetic state. **(A–F)** The diabetic wound manifests characteristics of impaired re-epithelialization **(A)**, aberrant angiogenesis **(B)**, and impaired collagen deposition **(C,D)** examined by Masson trichrome staining. Decreased extracellular matrix deposition **(E,F)** shown by H&E observation. **(G,H)** HIF-1α significantly decreased in diabetic wound. HIF-1α of normal and diabetic wounds was examined through immunohistochemistry. The collagen deposition density, extracellular matrix thickness, and HIF-1α positive area were calculated by ImageJ. Data are shown as mean ± SD. **p < 0.01, *n* = 3.

Compared with normal wounds, diabetic wounds showed an obviously delayed wound healing feature. As shown in Figure 1A, the reepithelialization of diabetic wounds was significantly slower than that of normal wounds. On the 11th day, when normal wounds show an almost completed reepithelialization, there still existed a large area of un-epithelialization in the diabetic wound. Meanwhile, the angiogenesis of diabetic wounds was also significantly disrupted in the diabetic state, and it was characterized as aberrant, with a leaky neovascular network and lower density compared with the normal wound on the fifth day (Figure 1B). The collagen deposition was examined by Masson trichrome staining, which showed lower collagen deposition in diabetic wounds (Figures 1C,D). The granular tissue representing the healing quality and extracellular matrix deposition also proved that the diabetic wound healing process was significantly impaired. The thickness of the granular tissue in the diabetic wound healing group was much lower than that of the normal group as shown in Figures 1E,F. As the main regulator of wound healing, HIF-1α was significantly unstable in diabetic wounds, which resulted in the non-healing feature of diabetic wounds. In the wounds of our diabetic model, we confirmed that HIF-1α was extremely unstable. As shown in Figures 1G,H, we found that the HIF-1α expression in the diabetic wound was significantly lower than that in normal wound.

### Insulin-Containing Wound Dressing Promotes Diabetic Wound Healing

**FIGURE 2:**
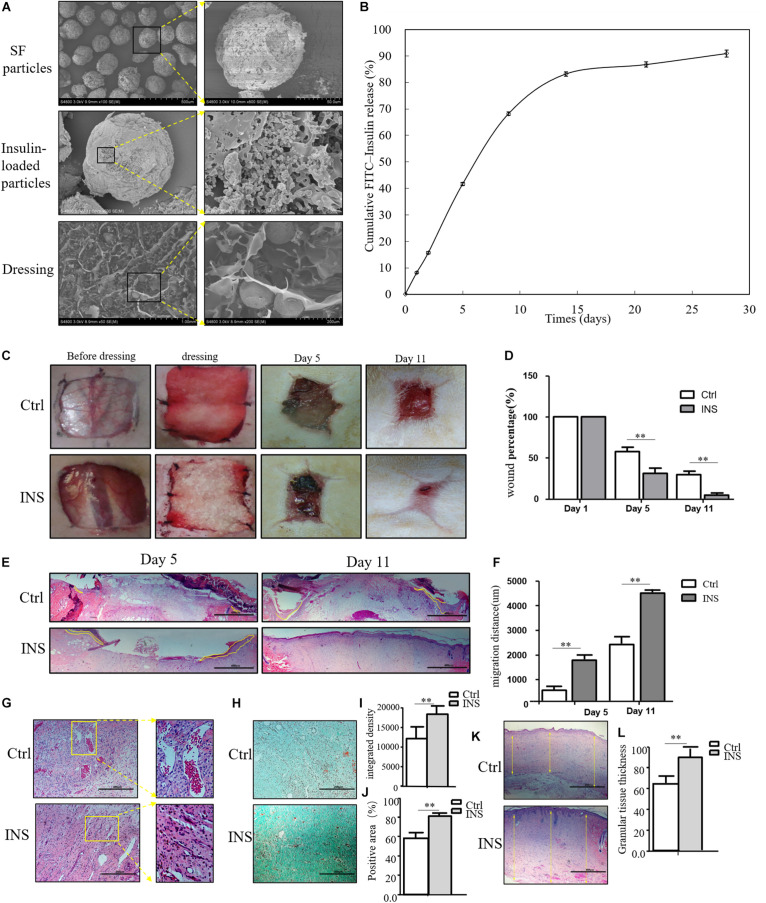
Insulin-containing SF microparticles promote diabetic wound healing. **(A)** The SEM and cross-sectional views of SF microparticles, insulin-encapsulated SF microparticles, and microparticle-loaded SF. **(B)** Cumulative FITC–insulin release from microparticle-loaded SF dressings. **(C,D)** Insulin promoted healing of diabetic wound. After the wounding procedure, the wounds in the control group (Ctrl) were dressed with SF microparticles without insulin; wounds were dressed with the insulin-containing SF microparticles in the insulin group (INS). Wound sizes were recorded on the 1st, 5th, and 11th days after wounding using transparent tracing papers. The unhealed rate of wounds was calculated by Image Pro Plus. Data are shown as mean ± SD. **p < 0.01, *n* = 5. **(E,F)** Insulin-containing dressing enhances reepithelialization of diabetic wounds. Reepithelialization was analyzed on the 5th and 11th day of wounding. The migration length of the tongue was calculated by Image Pro Plus. Data are shown as mean ± SD. **p < 0.01, *n* = 5. **(G)** Insulin-containing dressing alleviates aberrant angiogenesis of diabetic wounds. Angiogenesis quality of the 5th-day wounds was evaluated by H&E observation. **(H–J)** Insulin-containing dressing promotes collagen deposition of diabetic wounds. Masson trichrome staining was used to check collagen deposition in the wound. The integrated density and positive area of collagen deposition were calculated, and data are shown as mean ± SD. **p < 0.01, *n* = 5. **(K,L)** The thickness of the granular tissue was used as a marker of ECM deposition. Data are shown as mean ± SD. **p < 0.01, *n* = 5.

Based on our previous research results, topical insulin application could accelerate wound healing, but it had some shortcomings such as the inconvenience of insulin daily application and disruption of glucose level. Based on these reasons, we composed insulin-loaded SF microparticles. The SEM characteristics of this material were shown in Figure 2A, and this material could continuously release insulin for more than 20 days (Figure 2B). In this work, it was investigated whether the material promoted diabetic wound healing. As shown in Figures 2C,D, the wound almost healed in the group using insulin-containing dressing (INS) on the 11th day, whereas the control SF microparticles group (Ctrl) still had a large area of non-healing wound. The migration tongues of wounds on the 5th and 11th days also proved that the reepithelialization in the INS group was significantly faster than that in the Ctrl group in Figures 2E,F. Furthermore, the aberrant angiogenesis process in diabetic wounds was also improved in the INS group. Compared with the Ctrl group with the abnormally large and leaky neovascular network, the INS group had normal lumen and healthy vascular network as shown in Figure 2G. The impaired collagen deposition and extracellular matrix deposition were also ameliorated by insulin application as shown in Figures 2H–L.

### MGO Accumulated in the Diabetic State and Played a Toxic Effect on Wound Healing-Related Cells

**FIGURE 3:**
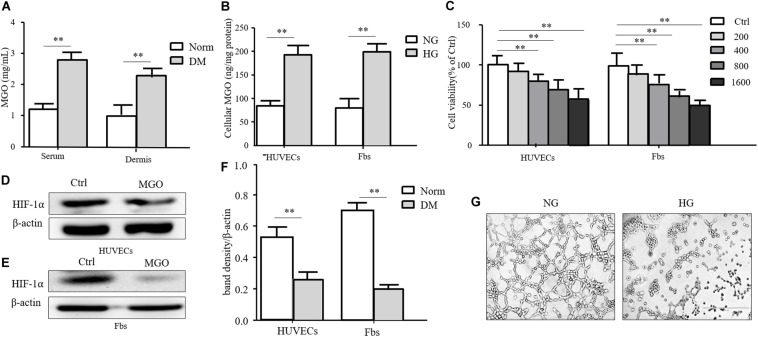
MGO accumulated in the diabetic state and induced an HIF-1α unstable state. **(A)** MGO accumulated in the diabetic state. Eight weeks after diabetic induction, the blood and dorsal dermis of diabetic (DM) and normal (Norm) rats were collected. The serum and dermis homogenate were subjected to MGO analysis. Data are shown as mean ± SD. **p < 0.01, *n* = 3. **(B)** High glucose incubation leads to MGO accumulation in HUVECs and fibroblasts. Five days after normal glucose (5.5 mM, NG), HUVECs and fibroblasts treated with high glucose (25 mM, HG) were harvested and lysed. The lysis was subjected to MGO analysis. Data are shown as mean ± SD. **p < 0.01, *n* = 3. **(C)** MGO impaired HUVEC and fibroblast viability in a concentration-dependent manner. HUVECs and fibroblasts were separately treated with 0 (control group), 200, 400, 800, and 1,600 μM MGO, and after 24 h of incubation, cells were harvested and subjected to CCK-8 analysis. The viability of each group was compared with that of the control group and presented as the percentage of the control group. Data are shown as mean ± SD. **p < 0.01, *n* = 3. **(D–F)** MGO induced unstable HIF-1α in the hypoxia state. After treatment with/without 800 μM MGO, HUVECs and fibroblasts were subjected to hypoxia (1% oxygen concentration) treatment. Twenty-four hours after treatment, cells were harvested and lysed. The lysis was subjected to western blot analysis for the HIF-1α level. Photoshop was used in the quantification of immunoblots. Data are shown as mean ± SD. **p < 0.01, *n* = 3. **(G)** MGO impaired angiogenesis. HUVECs were seeded into a Matrigel with/without 800 μM MGO for tube formation analysis. Twelve hours later, the tube formation status in both groups was photographed.

Insulin-containing dressing could promote diabetic wound healing, and its mechanism might relate to an improved HIF-1α signal pathway. However, whether insulin affected the HIF-1α signal pathway to improve the diabetic state is still unknown. In the diabetic state, many factors influenced the stabilization of HIF-1α, and the accumulation of AGEs was regarded as an indispensable one. MGO was the major form of AGEs and accumulated in the serum and tissue homogenate. As shown in Figure 3A, the MGO level was significantly higher in both serum and dermis in DM than in Norm. We also analyzed the MGO level in high-glucose cultured HUVECs and fibroblasts, which suggested that a higher glucose culture resulted in a higher MGO accumulation in both kinds of cells shown in Figure 3B. Based on the serum MGO level with different concentrations, it is found that MGO inhibited HUVEC and fibroblast proliferation in a concentration-dependent manner. In detail, 800 μM of MGO was sufficient to induce the vicious effects on HUVECs and fibroblasts as shown in Figure 3C. Therefore, 800 μM of MGO, as optimal concentration, could significantly destabilize HIF-1α under the hypoxia state in both HUVECs and fibroblasts as shown in Figures 3D–F. Meanwhile, 800 μM of MGO also exhibited the effect of inhibiting HUVEC tube formation as shown Figure 3G.

### Insulin-Containing Dressing Promotes Diabetic Wound Healing by Stabilizing HIF-1α

**FIGURE 4:**
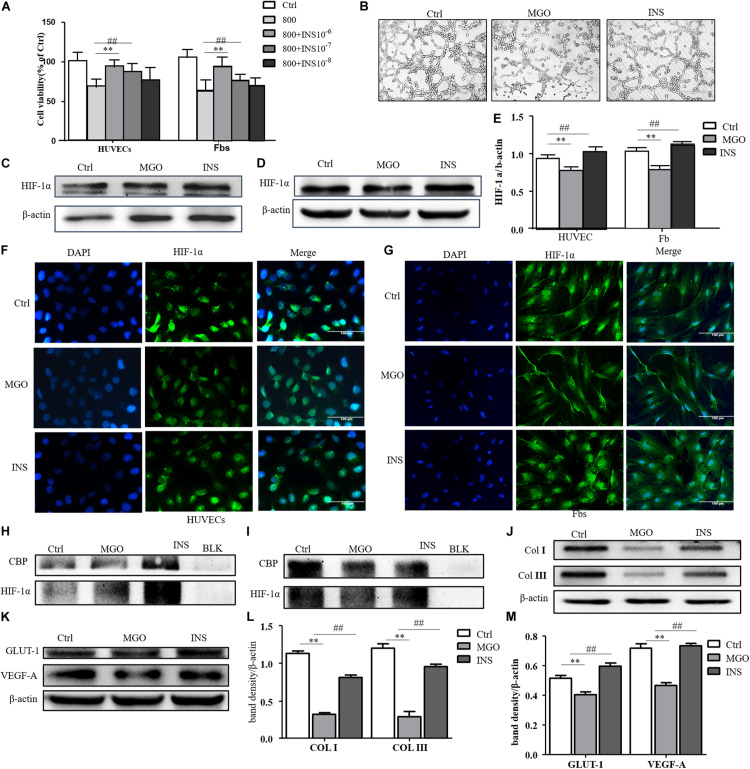
Insulin relieved MGO-induced HIF-1α impairment in wound healing cells. **(A)** Insulin promoted MGO-induced cell viability impairment in a concentration-dependent manner. HUVECs and fibroblasts were pretreated with 0 (control group) and 800 μM MGO for 30 min. Thirty minutes after MGO treatment, 0, 10^– 6^, 10^– 7^, and 10^– 8^ M insulin were added to the culture medium. After 24 h of incubation, cells were subjected to CCK-8 analysis. The viability of each group was compared with that of the control group and presented as the percentage of the control group. Data are shown as mean ± SD. **/##*p* < 0.01, *n* = 3. **(B)** Insulin promoted MGO-induced HUVEC angiogenesis impairment. HUVECs were set into three groups: control group (Ctrl) treated without MGO and insulin; MGO group (MGO) treated with 800 μM MGO; insulin group (INS) treated with 800 μM MGO and 10^– 6^ M insulin. The treatment was described previously. Twelve hours later, the tube formation status in both groups was photographed. **(C–E)** Insulin alleviated MGO-induced HIF-1α accumulation impairment of HUVECs and fibroblasts. HUVECs and fibroblasts were set as Ctrl, MGO, and INS groups and treated as described above. Twenty-four hours under the hypoxia state (1% oxygen concentration), cells were harvested, and HIF-1α levels were analyzed. Photoshop was used in the quantification of immunoblots. Data are shown as mean ± SD. **/##*p* < 0.01, *n* = 3. *Means Ctrl vs. MGO; #means MGO vs. INS. **(F,G)**. Insulin promoted HIF-1α translocation into HUVECs and fibroblasts. In Ctrl, MGO, and INS groups and after 24 h under the hypoxia state, cells were harvested and fixed with 4% paraformaldehyde. The cells were then subjected to immunofluorescence examination to analyze the HIF-1α accumulation and translocation to the nucleus. **(H,I)** Insulin promoted translation complex formation of HIF-1α and CBP in HUVECs and fibroblasts. Cell lysis of Ctrl, MGO, and INS was subjected to co-IP examination to analyze the HIF-1α and CBP complex formation. **(J–M)** Insulin improved MGO-induced HIF-1α pathway impairment in HUVECs and fibroblasts. Cell lysis of Ctrl, MGO, and INS was subjected to western blot to examine the expression of HIF-1α target genes VEGF-A and GLUT-1 (HUVECs) and collagen I and collagen III (fibroblasts). Photoshop was used in the quantification of immunoblots. Data are shown as mean ± SD. **/##*p* < 0.01, *n* = 5. *Means Ctrl vs. MGO; #means MGO vs. INS.

We next investigate whether insulin application could revise these impairments caused by MGO. The results showed that insulin could revise inhibitions of HUVEC and fibroblast proliferation in a concentration-dependent manner as shown in Figure 4A. Meanwhile, 10^–6^ mM of insulin, as optimal concentration, could significantly alleviate MGO-induced HUVEC tube formation impairment as shown in Figure 4B. Furthermore, the instability of HIF-1α was also significantly revised in the insulin application group through immunofluorescence. In detail, co-IP of HIF-1α and CBP, the signature of HIF-1α, exerted their transcription functions (Figures 4C–I), and protein expression of HIF-1α target genes was significantly enhanced as shown in Figures 4J–M. The results showed that insulin promoted HIF-1α stabilization by the translocating into the nucleus.

### Insulin-Containing Material Promotes Wound Healing Through HIF-1α and Its Downstream Effector

**FIGURE 5:**
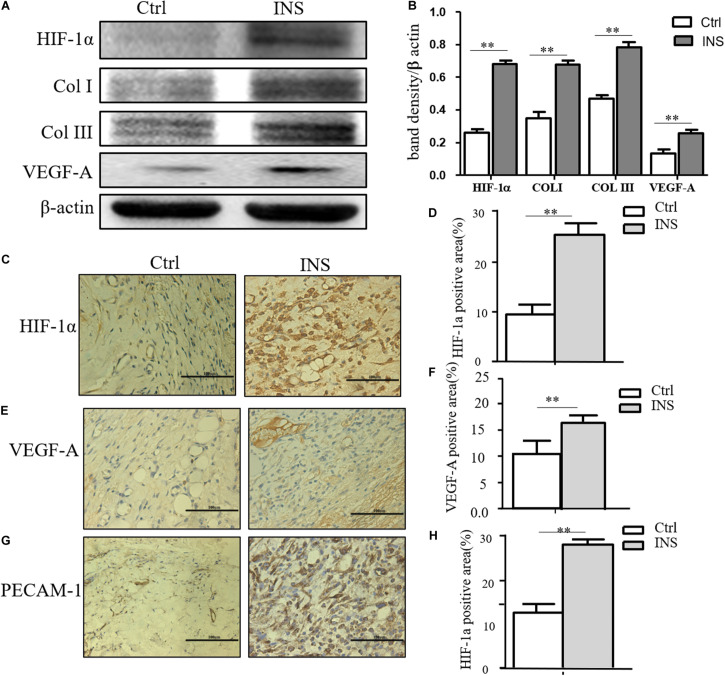
Insulin-containing SF microparticles promoted diabetic wound healing through HIF-1α stabilization. **(A,B)** Insulin-containing dressing promoted HIF-1α stabilization and its downstream protein expression. The expressions of HIF-1α and downstream protein Col I, Col III, and VEGF-A in wounds on the fifth day were analyzed using western blot, and Photoshop was used in the quantification of immunoblots. Data are shown as mean ± SD. **p < 0.01, *n* = 5. **(C–F)** Immunohistochemistry examination of HIF-1α and VEGF-A expressions and the positive area of the stain were calculated by ImageJ. Data are shown as mean ± SD. **p < 0.01, *n* = 5. **(G,H)** Immunohistochemistry examination of angiogenesis in both groups. PECAM-1 was used as the marker of endothelial cells in rat vessels, and the positive area of the stain was calculated by ImageJ. Data are shown as mean ± SD. **p < 0.01, *n* = 5.

We confirmed in this work that insulin application could reverse MGO-induced HIF-1α instability and promote its signal effect, an effect of insulin-containing material treating wounds. HIF-1α and its downstream effector including VEGF-A, GLUT-1, Col I, and Col III were significantly higher in insulin-containing dressing wounds as shown in Figures 5A,B. The immunochemistry examination of VEGF-A, vessel density, and collagen deposition further proved this assumption (Figures 5C,D).

## Discussion

Once a wound formed, the healing process initiates. Due to the destruction of blood supply and the high proliferation potential of wound cells, the wound is in an absolute or relative hypoxia state during a considerable part of the time. The process of wound healing in other words is the recovery of tissue structure and oxygen supply. HIF-1α plays a very important role in this process and is constitutively expressed in most cells. However, it is very sensitive to oxygen and would be degraded by the ubiquitin-proteasome immediately under a normoxia state. Under the hypoxia state in the wound healing environment, HIF-1α-degrading pathways are inhibited, and HIF-1α accumulates in the cytoplasm due to the inhibited HIF-1α-degrading pathways, and then it translocates into the nucleus to exert its translation factor function. When forming a transcription complex with HIF-1β, the co-activator CBP initiates a number of gene expression targets to inflammation reaction, angiogenesis, reepithelialization, and extracellular matrix deposition (Zhao et al., 2012). Under the diabetic state, high glucose and its side-products can inhibit the stability of HIF-1α in the hypoxia environment in many ways. MGO is a by-product of cell glucose metabolism in the condition of diabetes mellitus and has a significantly increased concentration in both rats and high-glucose cultured cells. Results showed that MGO can inhibit HIF-1α signal pathways in many ways such as downregulating the stability of HIF-1α through enhancing the chaperone-dependent ubiquitin degradation of HIF-1α (Bento et al., 2010), promoting the activity of PHD activation, and influencing the combination of HIF-1α and HIF-1β or HIF-1α and CBP (Bento and Pereira, 2011). Our results show that MGO affects the stability of HIF-1α and decreases the binding of HIF-1α and CBP. The significantly decreased protein levels of HIF-1α and its downstream target genes after MGO treatment further proved the vicious role that MGO plays in the HIF-1α-mediated wound healing process. The impairment of the HIF-1α pathway was considered as a pivotal mechanism of diabetic non-healing wounds. Promoting the stability of HIF-1α is an important research direction targeting at promotion of diabetic wound healing (Catrina and Zheng, 2016). Our previous researches confirm that insulin can promote wound healing by promoting epidermal migration, angiogenesis, extracellular matrix deposition, etc., which are exactly the downstream effects of HIF-1α. These results focus on HIF-1α and its downstream pathway. The application of insulin can promote the accumulation of HIF-1α in the cytoplasm and nucleus, and then HIF-1α will combine with CBP to activate its downstream transcription. In summary, this study confirmed that the increase of MGO content in diabetes can lead to the instability of HIF-1α, which results in the delay of wound healing due to the damage of the HIF-1α signal pathway and its downstream effects. Wound dressing containing insulin can stabilize HIF-1α, promote the downstream effects related to HIF-1α, and enhance the healing of diabetic wound.

## Data Availability Statement

The raw data supporting the conclusions of this article will be made available by the authors, without undue reservation.

## Ethics Statement

The animal study was reviewed and approved by Animal Care Committee of SJTUSM.

## Author Contributions

All authors listed have made a substantial, direct and intellectual contribution to the work, and approved it for publication.

## Conflict of Interest

The authors declare that the research was conducted in the absence of any commercial or financial relationships that could be construed as a potential conflict of interest.
